# Dietary Intakes of Professional and Semi-Professional Team Sport Athletes Do Not Meet Sport Nutrition Recommendations—A Systematic Literature Review

**DOI:** 10.3390/nu11051160

**Published:** 2019-05-23

**Authors:** Sarah L. Jenner, Georgina L. Buckley, Regina Belski, Brooke L. Devlin, Adrienne K. Forsyth

**Affiliations:** 1 Department of Rehabilitation, Nutrition and Sport, La Trobe University, Bundoora, VIC 3068, Australia; B.Devlin@latrobe.edu.au (B.L.D.); A.Forsyth@latrobe.edu.au (A.K.F.); 2 Carlton Football Club, Ikon Park, Carlton, VIC 3053, Australia; 3 School of Health Sciences, Swinburne University of Technology, Hawthorn, VIC 3122, Australia; gbuckley@swin.edu.au (G.L.B.); rbelski@swin.edu.au (R.B.)

**Keywords:** sports nutrition, carbohydrate intake, energy, nutritional recommendations

## Abstract

Background: to develop sport-specific and effective dietary advice, it is important to understand the dietary intakes of team sport athletes. This systematic literature review aims to (1) assess the dietary intakes of professional and semi-professional team sport athletes and (2) to identify priority areas for dietetic intervention. Methods: an extensive search of MEDLINE, Sports DISCUS, CINAHL, Web of Science, and Scopus databases in April–May 2018 was conducted and identified 646 studies. Included studies recruited team sport, competitive (i.e., professional or semi-professional) athletes over the age of 18 years. An assessment of dietary intake in studies was required and due to the variability of data (i.e., nutrient and food group data) a meta-analysis was not undertaken. Two independent authors extracted data using a standardised process. Results: 21 (*n* = 511) studies that assessed dietary intake of team sport athletes met the inclusion criteria. Most reported that professional and semi-professional athletes’ dietary intakes met or exceeded recommendations during training and competition for protein and/or fat, but not energy and carbohydrate. Limitations in articles include small sample sizes, heterogeneity of data and existence of underreporting. Conclusions: this review highlights the need for sport-specific dietary recommendations that focus on energy and carbohydrate intake. Further exploration of factors influencing athletes’ dietary intakes including why athletes’ dietary intakes do not meet energy and/or carbohydrate recommendations is required.

## 1. Introduction

### 1.1. Nutrition for Team Sport Athletes

Team sports can be defined as field- and court-based sports with intermittent and high-intensity game patterns [1]. Match patterns will vary markedly among different sports and for specific positions within a sport. Team sports are divided into three categories: (1) endurance-based sports including football (i.e., soccer), Australian football, hockey (2) strength and power sports such as rugby union and league, American football and (3) batting sports such as baseball, softball and cricket [1]. Athletes participating in professional team sports are supported by high-performance and medical staff, which aim to monitor and optimise fitness, body composition and performance outcomes. The physiological demands of team sports differ and can include a range of performance modes including: running moderate to long distances, high intensity bouts of movement, variable activity patterns and small bouts of rest periods [1,2,3]. The variable nature of team sport exercise requires use both anaerobic and aerobic systems to fuel performance [1]. Therefore, each team sport and position within the sport, depending on the nature of training and competition, will have unique energy demands and nutrient requirements. To optimise performance and enhance recovery, international sporting committees (i.e., International Olympic Committee (IOC), American College of Sports Medicine (ACSM), International Society of Sports Nutrition (ISSN)) have provided nutrition recommendations to support dietitians working with athletes to meet their individual nutrition needs [4,5,6,7,8,9].

Dietitians must consider a range of sport-specific factors including the rules, arena size, timing of competition, frequency of matches and length of seasons (including macrocycles: preseason, competition season, off-season) when assessing an athlete’s nutrition requirements and goals. Additionally, the physique characteristics and position-specific tasks of the sport will further influence the nutritional requirements of athletes. For example, the sport of rugby union will require forwards to be heavier and stronger in comparison to backs who need to be leaner and faster [10]. Due to the sport-specific factors, physique and position differences, dietary advice for team sport athletes should be individualised.

Recommendations that support athletes to consume sufficient energy and the correct balance of macronutrients and micronutrients, with appropriate timing to enhance performance and recovery, will enable athletes to train and perform optimally [11]. An earlier review by Holway and Spriet [1] found that athletes competing in team sports commonly do not meet recommended dietary intake needs [1,12]. Those that fail to consume energy and/or maintain a diet that encompasses the appropriate balance of macronutrients may find that this impedes on training adaptations and recovery [11,12]. Deficiencies in energy can have implications for an athlete’s performance including a loss of fat free mass, disturbances to immune function, decreased bone mineral density, increased susceptibility to injury and increased prevalence of symptoms of overtraining [11].

### 1.2. Objectives

In the past decade, thousands of new research papers have been published in sports nutrition and 17 new consensus statements and recommendation papers have been released by authoritative organisations such as the IOC, ACSM, ISSN [4,5,6,7,8,9,11,13,14,15,16,17,18,19,20,21,22]. There have also been a large number of published studies on the dietary intake of professional and semi-professional team sport athletes during this time [2,3,10,23,24,25,26,27,28,29,30,31,32,33,34,35,36,37,38,39]. With new sports nutrition recommendations [5,6,7,8,11,13,14,15,16,17,18,19,20,21] and updated literature reporting the dietary intake of team sport athletes, it is now timely to review the literature to determine whether team sport athletes consume diets that align with the sports nutrition recommendations [2,3,10,23,24,25,26,27,28,29,30,31,32,33,34,35,36,37,38,39]. This paper aims to review the literature on dietary intakes of professional and semi-professional team sport athletes systematically with the aim of identifying priority areas for dietetic intervention.

## 2. Materials and Methods

### 2.1. Protocol Registration

All methods and search strategies were aligned with Preferred Reporting Items for Systematic Review (PRISMA) guidelines. This review was registered with International Prospective Register of Systematic Reviews (registration number: CRD42018105168) [40,41]. A PICOS criteria (i.e., Participants, Intervention, Comparison, Outcome and Study design) for review is defined in Table 1. A systematic search using terms such as sport or team sport or dietary intake or food intake was conducted by one researcher (SJ). All keywords used in search are listed in Table 2.

### 2.2. Search Strategy

Five electronic databases including MEDLINE (Medlars International Literature Online), Sports DISCUS, CINAHL (Cumulative Index of Nursing and Allied Health Literature), Web of Science (WOS), and Scopus (World’s largest abstract and citation database of peer-reviewed literature) were searched to investigate the dietary intake of professional and semi-professional team sport athletes by one researcher (SJ). The timeframe designated for the search included studies published from 2011 to present (i.e., after the 2011 review by Holway and Spriet) [1]. An additional limit regarding age (i.e., >18 years) was included to limit results to adult athletes only. In order to identify any further relevant publications, the reference lists of the studies included were hand searched and other manual searches were conducted (i.e., Google Scholar).

### 2.3. Eligibility Criteria

All original research (i.e., cross-sectional, longitudinal, published and unpublished thesis) conducted in adult team sport athletes (i.e., 18 years and older) and published since 2011 was considered for inclusion (Table 3). Randomised control trials were additionally included in the review if baseline dietary intake data was available. Randomised control trials where only post intervention dietary intakes were available, conference posters, abstracts and web-based articles were not included for review. Only professional and semi-professional athletes were included in the review; amateur and recreational athletes were not included. Only English-language studies were included for this review. Included studies were required to provide baseline or habitual dietary intake data that quantified energy, macronutrients and micronutrients to allow for the specified conversions made and displayed in the data extraction table i.e., energy (MJ/day), carbohydrate (grams (g) and g·kg^−1^·day^−1^), protein (g and g·kg^−1^·day^−1^), fat (g and % total energy), calcium (mg/day) and iron (mg/day). Studies that reported dietary habits, dietary knowledge, attitudes, and education strategies where dietary intake was not quantified, were excluded from the review.

### 2.4. Study Selection

**Chart 1:**
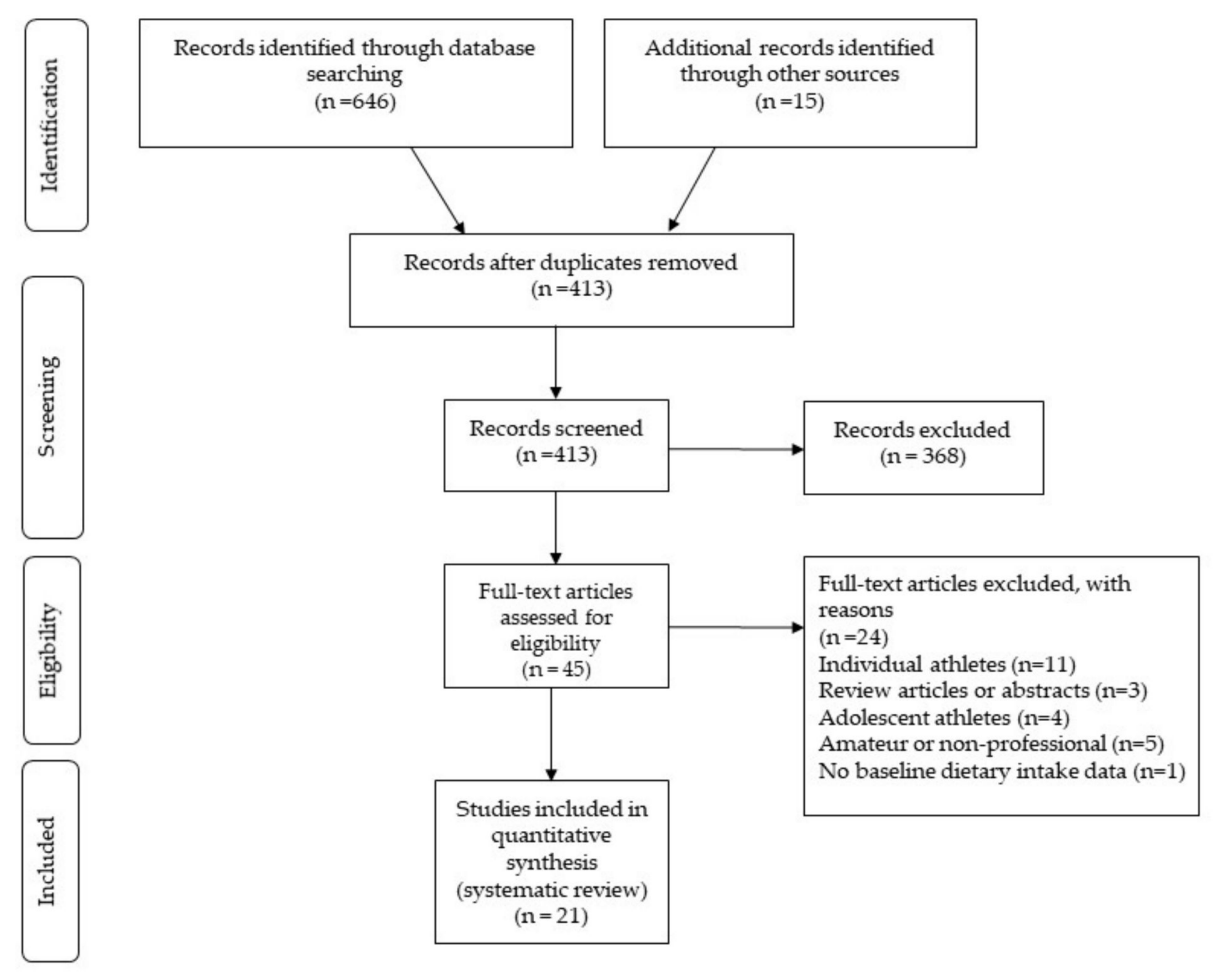
Study section process.

All studies were screened based on title and abstract by main author (SJ). Articles deemed eligible for full text review were screened against inclusion and exclusion criteria (Table 3) by two authors (SJ and GB). Additional reviewers (AF and RB) provided advice on eligibility if a decision for inclusion and exclusion required feedback. Selection of included studies and reasons for exclusion are reported in Chart 1.

### 2.5. Data Collection Process

Dietary intake data were extracted from the included studies by two authors (SJ and GB). Data presented in Table 4 include participant demographics including sport, anthropometry measures (total mass (kg), body fat (%), fat free mass (kg)) and athletic level (professional or semi-professional)); details regarding the study background and methods; country of origin and survey method (i.e., 7-day food diary) and dietary intake results including total energy/calories (i.e., MJ), carbohydrate intake (i.e., g and g·kg^−1^·day^−1^), protein intake (i.e., g and g·kg^−1^·day^−1^), fat intake (i.e., g and % total energy), calcium intake (i.e., mg) and iron intake (mg). Where total energy intake was reported in calories, this was converted to MJ to enable comparison across studies.

### 2.6. Study Quality: Risk of Bias

The quality of studies was examined by two authors (SJ and GB) using the Academy of Nutrition and Dietetics Quality Criteria Checklist from the Academy of Nutrition and Dietetics Evidence Analysis Manual [42]. The quality criteria checklist provides an assessment based on relevance and validity criteria questions, ranking studies as either positive, neutral or negative. A third reviewer (AF) reviewed any discrepancies that occurred during the quality analysis. Studies with positive ratings needed to describe study selection adequately (including inclusion and exclusion criteria), methods of comparing groups and the study setting, and include measurements that were valid and reliable.

## 3. Results

### 3.1. Study Selection

The original search retrieved 646 studies that fit the search criteria with an additional 15 studies identified by hand searches (Chart 1). After duplicates were removed, a title and abstract exclusion was undertaken and 45 studies were retained for full text assessment. After completion of full text assessment 21 studies were included in this review for data extraction, quality assessment and analysis. All studies included in the review had a positive or neutral quality rating [41].

### 3.2. Study Characteristics

The majority of studies included in this review included team sport athletes competing professionally and semi-professionally in Australia (*n* = 255) [3,25,26,33,43] and Spain (*n* = 81) [28,29,30,36], with the remaining studies including athletes from Europe (not-specified) (*n* = 34) [10,27], England (*n* = 30) [23,34], America (*n* = 26) [32,38], Canada (*n* = 25) [39], Brazil (*n* = 19) [37], Netherlands (*n* = 14) [24], South Africa (*n* = 11) [35], United Kingdom (*n* = 10) [2], and Mexico (*n* = 6) [31,37]. The majority of studies included in this review reported the dietary intakes of professional team sport athletes [2,3,10,23,24,26,27,28,29,31,33,34,36,37], with additional studies exploring dietary intake of semi-professional team sport athletes [32,35,38,39] and studies exploring the dietary intakes of a combination of sports [25,30,43]. Studies included a range of team sport athletes with the majority of studies reporting on the dietary intakes of football athletes (*n* = 210) [23,24,25,28,30,31,34,37], followed by Australian football (*n* = 139) [3,26,43]. Across the remaining studies, other team sport athletes represented in this review include; rugby union (*n* = 70) [10,27,33,35], ice-hockey (*n* = 25) [39], wheelchair basketball (*n* = 17) [29], American football (*n* = 15) [32], handball (*n* = 14) [36], volleyball (*n* = 11) [38], rugby league (*n* = 10) [2].

The majority of studies included in this review used a cross-sectional study design (*n* = 14), with the remaining studies using pre-post-test [32,38], case-study [27,29], case control [30] and longitudinal [26,36] designs to assess dietary intake. Dietary intake data were collected most frequently using food diaries/records (weighed and not weighed). Studies used 7-day food diaries [2,3,23,28,33,35,39], 3-day food diaries [29,32,37,38] and 24 hr recalls [10,24,25,43]. Other studies used food diaries of different duration (i.e., 4-day, 6-day, and 8-day) [27,30,34], dietary recalls (i.e., 72-h) [26,36] and a combination of 4-day weighed food diary in addition to a food frequency questionnaire (FFQ) to assess dietary intake [31].

### 3.3. Dietary Intake of Team Sport Athletes

#### 3.3.1. Energy Intake of Team Sport Athletes on Training Days

All studies included assessed the energy intake of team sport athletes. The majority of authors assessed the energy intake of athletes against recommendations advocated by IOC, ACSM, ISSN and sports specific research [1,4,12,35,44,45,46,47,48,49,50,51,52,53,54,55]. Several studies provided evidence that energy intake of team sport athletes assessed was suboptimal and did not meet recommendations [2,3,10,26,28,35,36,38]. Of the 21 studies included, five studies reported that energy intake was adequate according to the respective dietary recommendations used [23,32,33,37,39]. One study by Devlin et al. [43] reported that the Australian football athletes included met energy recommendations, however the football athletes did not. The remaining studies (*n* = 7) did not report on adequacy of energy intake (i.e., recommendations met versus not met) [24,25,27,29,30,31,34].

#### 3.3.2. Energy Intake of Team Sport Athletes during Competition

The mean energy intake reported in this review ranged from 9.1–16.6 MJ/day and 7.3–9.8 MJ/day for males and females respectively. Seven studies included comparison data of dietary intake on training days and match days [2,23,24,27,31,35,39]. Two studies that explored the dietary intake of rugby union (*n* = 10) and ice hockey (*n* = 25) athletes on match days found that energy intake did not meet increased requirements for the fueling and recovery required on match days [2,39]. However, in comparison, two studies that included professional rugby union (*n* = 14) and football athletes (*n* = 6), found that energy intake was greater on match days in comparison to training days [23,27]. In particular, research by Bradley et al. [27] found that on average professional rugby union athletes increased their energy intake in preparation for game day in comparison to the first four days of training where energy intake was reduced, irrespective of energy expenditure. Anderson et al. [23] additionally found that professional football players (*n* = 6) had a greater absolute and relative energy intake on match days in comparison to training days.

#### 3.3.3. Macronutrient Intake of Team Sport Athletes on Training Days

Overall, the macronutrient composition of the diets of team sport athletes was inadequate to meet the fuel, recovery and performance demands of their sports. All but one study assessed the dietary intake of carbohydrate and protein [30] and all but two assessed the dietary intake of fat [30,34]. Overall, a macronutrient imbalance was found in the majority of studies with most athletes reported consuming diets high in protein and fat, at the expense of carbohydrate. Team sport athletes including athletes from football (*n* = 175), Australian football (*n* = 139), rugby union (*n* = 88), volleyball (*n* = 11) and rugby league (*n* = 10) consumed diets that consistently did not meet carbohydrate recommendations [2,3,10,23,24,25,26,27,28,30,33,35,36,37,38,43]. Out of the 17 studies that provided mean intakes of carbohydrates, 15 reported low carbohydrate intake and fell below ISSN recommended intakes of 5-8 g·kg^−1^·day^−1^ (range: 2.4–4.9 g·kg^−1^·day^−1^ and 3.08–4.6 g·kg^−1^·day^−1^ for male and female athletes respectively). One study that explored the dietary intakes of male professional football players found that carbohydrate intakes consumed by athletes were closer to meeting recommendations for tactical or skill based sports (3–5 g·kg^−1^·day^−1^) [12,25].

Conversely, the majority of studies found that dietary intake of protein [2,10,24,25,26,28,30,31,33,35,36,37,43,56] and fat [24,27,28,32,35,36,37] exceeded recommendations. Eight studies that included athletes competing professionally and semi-professionally in Australian football, rugby league, rugby union and football found protein intakes in excess of 2.0 g·kg^−1^·day^−1^ [2,10,25,27,31,33,35,43], with a study reporting that the diets of professional Australian football athletes on average contained 3.4 ± 1.1 g·kg^−1^·day^−1^ of protein per day [43]. Studies included in this review reported dietary intakes that were high in protein, however low in carbohydrates and/or total energy (hypocaloric) [2,3,10,26,28,35,36,38,43]. Nine studies found that dietary intake of fat exceeded recommendations [2,27,28,29,31,32,35,37,39]. Three studies that included rugby union athletes (*n* = 35) and football athletes (*n* = 25) found that while overall total fat intake exceeded recommendations, polyunsaturated fat intake fell below recommended intakes of 10% of total energy [31,35,37]. Six studies explored the intake of cholesterol, finding that dietary intakes of athletes exceeded recommended intakes of <300 mg/day [2,28,31,32,35,37]. Kirwan et al. [32] linked high cholesterol intakes to potential body composition goals of American football athletes (i.e., to put on mass, quickly).

#### 3.3.4. Macronutrient Intake of Team Sport Athletes during Competition

Three of seven studies that assessed intake on competition days found that carbohydrate consumed in preparation for match day, during the match and during the recovery period post game, did not meet recommended intakes for competitions [24,31,39]. One study in female ice-hockey players found that there was no significant difference in carbohydrate, protein and fat intakes between game, training and rest days [39]. In comparison, three studies on rugby union (2) and football (1) athletes found that carbohydrate intake during and post-game day met recommendations [23,27,35]. In particular, Anderson et al. [23] found that athletes practiced a level of periodization, finding that carbohydrate consumed on the two match days were significantly greater than carbohydrate consumed on training days (*p* = < 0.05; 6.4 g·kg^−1^·day^−1^ and 4.2 g·kg^−1^·day^−1^ for match and training days respectively). In comparison, research by Bettonviel et al. [24] found that professional football players as a whole failed to meet carbohydrate recommendations on match days (5.3 ± 1.5 g·kg^−1^·day^−1^) and one day post-match (4.5 ± 1.0 g·kg^−1^·day^−1^); however, protein intakes on match day and post-match were adequate (2.0 ± 0.4 and 1.6 ± 0.3 g·kg^−1^·day^−1^ respectively).

#### 3.3.5. Micronutrient Intake

Six studies reported iron and/or calcium intakes [3,27,28,35,36,37,39]. The majority of these studies found that team sport athletes were meeting or exceeding recommended intakes of calcium and iron, when compared to the general public [27,28,35,36,39]. One study by Raizel et al. [37] found that professional football players were not meeting general (non-sport specific) recommendations (estimated average requirements) for calcium, reporting that athletes had a marginal intake of 83% of EAR.

## 4. Discussion

### 4.1. Energy Intake of Team Sport Athletes

In order for athletes to optimise training and performance, they need to consume sufficient energy for the work required and to support physiological adaptions [11,13,57]. A diet that contains insufficient energy (i.e., energy deficit) during periods of training can result in a number of performance detriments including loss of lean muscle mass and bone mineral density, increased prevalence of overtraining and injury, and may contribute to endocrine and reproductive system disturbances [11]. The mean energy intakes of professional male team sport athletes reported in the literature have decreased from those reported by a previous review [1]; however, in comparison female athletes are consuming diets of relatively similar energy density (i.e., 7.3–9.8 MJ/day) [1]. Simply looking at the difference in energy intakes of male team athletes would suggest that they are eating less and potentially not meeting energy recommendations; however, a range of factors may have influenced the dietary intake of these athletes at the time of assessment.

While not identified as an influencing factor previously [1], three studies included in this review that explored the dietary intakes of rugby union, Australian football and football athletes suggested that low energy intakes were related to the presence of team culture surrounding body composition goals (i.e., to decrease body fat and/or increase lean muscle mass) [3,10,25]. In theory, body composition goals should be individualised to the athlete, with the focus to support training adaptions; however, this is not always reflected in practice [8]. Research by Bradley et al. [10] found that during a rugby union preseason, athletes did not meet energy intake recommendations (14.8 ± 1.9 MJ/day) and it was found that the existence of body composition goals (i.e., to reduce body fat) were potentially influencing intake at the expense of fueling for training demands. Research during a preseason in Australian football, hypothesised that athletes intentionally restricted energy and carbohydrate intake surrounding body composition assessments using dual-energy X-ray absorptiometry (DXA), to meet target body composition goals (actual energy intake: 9.1 ± 1.8 MJ/day) [3]. Similarly, Andrews et al. [25] reported professional and semi-professional football athletes that were recognised as under reporters were those athletes that were identified as attempting to maintain body composition or reduce body fat at the time of recording (11.5 ± 2.0 and 10.8 ± 3.8 MJ/day). Realistic body composition goals must be promoted to prevent under fueling and support the training adaptions and recovery of athletes. Furthermore, in light of recent research regarding relative energy deficiency (RED-S) in male athletes, there is a greater need for education for coaches and support staff regarding the importance of an individualised approach when tailoring body composition goals [8].

### 4.2. Macronutrient Intake of Team Sport Athletes

#### 4.2.1. Carbohydrate Intake of Team Sport Athletes

Carbohydrate intake is important for optimising performance and recovery. Team sports have varied training and physiological demands, therefore advice must be tailored to match the training demands as well as the demands of specific positions within the sport. Team sport athletes included in this review continue to consume diets that do not align with carbohydrate recommendations, intakes on average insufficient when compared to recommendations [1,11,12,52]. Research included has suggested that carbohydrate recommendations need to be better suited to the demands of the sport, individualising nutrition based on positions [10,23,28,58]. Research by Bradley et al. [27] found that professional rugby union athletes tapered carbohydrate intake across a training week; however, on average intakes fell below recommendations used by authors of 6–10 g·kg^−1^·day^−1^ (3.5 g·kg^−1^·day^−1^ and 3.4 g·kg^−1^·day^−1^ for forwards and backs respectively). Although these intakes did not align with recommendations, Bradley et al. [27] suggested that the tapering of intakes during a training week to match demands of training as well as enhance training adaptions (i.e., alter body fat) may be better suited to this athletic population. The idea of “*fueling for the work required*” is commonly being used by many sports nutrition professionals working in professional sport [59]. The concept of periodising intake is not simple and requires thorough knowledge of the athlete’s needs, the sport and the training and competition demands. Anderson et al. [23] found that football athletes were applying the principle of carbohydrate periodization to their daily intakes; consuming significantly greater energy and carbohydrate on game days compared to training days. However, when assessing dietary intakes as a whole; carbohydrate intake the day before a competition was unlikely to maximise glycogen storage and, therefore, meet match demands. Dietitians working with professional team sport athletes need to use an individualised approach when periodising an athlete’s carbohydrate intake. Further work is required to support education in this space to optimise glycogen storage and resynthesis and to support athletes’ training and match day nutrition goals.

#### 4.2.2. Protein Intake of Team Sport Athletes

Protein is important for muscle protein synthesis, supports recovery processes, promotes satiety and can aid the maintenance of body composition. It also has many other important roles in the body as enzymes, hormones, transporters and antibodies. Athletes with insufficient protein intakes have an increased risk of muscle wasting, illness and injury. ISSN recommendations state that to maintain protein balance athletes should consume 1.4–2.0 g·kg^−1^·day^−1^ of high-quality protein [11]. In this review team sport athletes, on average, adequately met recommendations; most athletes adopting a diet high in protein, but low in carbohydrates and/or total energy (hypocaloric) [2,3,10,26,28,35,36,38,43]. Research has suggested that high-protein diets (2.3–3.1 g·kg^−1^·day^−1^) may be appropriate for resistance trained athletes who are consuming hypocaloric diets with the aim to maintain lean muscle tissue while reducing fat mass [20,60]. This is supported by research by Bradley et al. [10] who found that although protein intakes of professional rugby athletes (2.5–2.6 g·kg^−1^·day^−1^) exceeded protein recommendations, since athletes were manipulating carbohydrate intakes during the preseason training week, protein intakes may have been appropriate at the time to optimise body composition. Given the context of the time of season this study was undertaken (i.e., preseason) and the ability for athletes to optimise training adaptions and changes to body composition without any detriment to competition performance, these intakes may be acceptable for this athletic cohort. However, in contrast, research by Potgieter et al. [35] reported that rugby union athletes’ intakes in-season did not meet carbohydrate recommendations, and exceeded protein recommendations. Potgieter et al. [35] suggests that greater intakes of protein (i.e., 2.4 g·kg^−1^·day^−1^) may be suited in times where muscle hypertrophy is required (i.e., offseason); however, in-season where athletes are required to meet training, competition and recovery demands, protein intakes should align with recommendations and not be increased at the expense of carbohydrate intake. Similarly, research by Mackenzie et al. [33] on rugby union athletes reported that there was no compelling evidence to increase the distribution of protein for muscle protein synthesis and that an excess quantity of protein may in fact compromise lean muscle goals by promoting satiety which can result in decreased calorie intake. Taken as a whole, when working with team sport athletes greater emphasis should be placed on the distribution and timing of protein intake across a training day, instead of the total quantity [33]. In addition, it should be highlighted that the need to meet a body composition goal should not come at the expense of meeting nutrient requirements for performance and recovery.

#### 4.2.3. Fat Intake of Team Sport Athletes

Four of the studies that reported athletes’ diets exceeding recommended fat intakes (i.e., >30% of total energy) included the dietary intakes of athletes competing in strength and power sports such as rugby union (2), rugby league (1) and American football (1) [2,27,32,35]. Collectively strength and power sports are characterised as high-intensity and intermittent collision sports and may require a greater total mass to protect athletes against the physical impacts of scrumming, tackling etc. [1,2,27,32,35]. A study that explored the dietary intakes of American football athletes found that athletes consumed high intakes of fat (i.e., 41% of total energy) suggesting these intakes were a result of overfeeding to increase weight [32]. In comparison, research by Bradley et al. [27] in rugby union found that athletes had fat intakes that fell slightly above recommendations (i.e., 32% and 33% for forwards and backs respectively); however, in contrast they had suboptimal total energy intake when compared to energy expenditure. Additionally Potgieter et al. [35] found that competing rugby union athletes were consuming an excess in fat (i.e., 33.8 ± 4.3%), however failed to meet recommendations for total energy and carbohydrate. Taken together, these results indicate a reduction in dietary fat intake for these groups may not be warranted in order to support athletes to meet energy requirements. However, as many athletes did not meet these enhanced energy requirements, the addition of carbohydrate may allow athletes to meet energy balance or surplus, without the need for intakes that are in excess of dietary fat.

Although most team sport athletes included in this review consumed diets that were in line with or exceeded recommendations for dietary fat intake, the composition of saturated to unsaturated fats (i.e., mono and poly unsaturated fats) was suboptimal (i.e., saturated fat >10% total energy). This is supported by Hidalgo et al. [31] who found that football players’ total dietary fat intake aligned with recommendations (i.e., 30%–33% of total energy); however, saturated fat and cholesterol intake exceeded recommendations. In addition, it was found that polyunsaturated fat intake fell below recommended intakes. Hidalgo et al. [31] and Raizel et al. [37] together suggested that football athletes’ high protein intake, including a large intake of animal proteins, may have contributed to their overall high cholesterol intake. Research suggests diets that are rich in saturated fat (i.e., >10%TE), cholesterol and trans fats are linked to chronic diseases such as cardio-vascular disease (i.e., heart disease and stroke) and type 2 diabetes [61]. For the long term health of athletes, dietary advice should aim to include a varied diet with a focus on total and saturated fat, not exceeding recommendations (i.e., total fat <30% total energy and saturated fat <10% total energy) [11]. Additionally, athletes may benefit from the inclusion of mono- and poly unsaturated fat-based protein foods (i.e., fatty fish, nuts and seeds) to help meet energy and protein requirements and provide additional anti-inflammatory benefits for training and recovery [22].

This review highlights the role of dietitians in providing long-term dietary strategies to increase lean muscle and total body mass, in a manner that does not adversely affect performance and/or lipid profile, which may be evident in short term high energy and high fat diets. Greater education regarding the long-term implications of intakes that are in excess of total fat and saturated fat is required in team sport environments.

#### 4.2.4. Dietary Intake during Competition

Due to the elevated requirements for stored glycogen and glycogen resynthesis during training and competition, athletes are recommended to undertake aggressive carbohydrate feeding prior to these periods. Inconsistencies between energy and carbohydrate intakes on training and match days were observed in team sport athletes. In particular research by Bettonviel et al. [24] found that carbohydrate intakes of football athletes did not meet recommendations (6–10 g·kg^−1^·day^−1^) for match days; however, in comparison these athletes exceeded recommended protein intakes. Additionally, research by Potgieter et al. [35] found that while rugby union athletes were consuming adequate energy and carbohydrate prior to competition, diets were additionally high in protein and fat. Interestingly, research by Tooley et al. [2] found that rugby league athletes had greater fat intakes on the two days post competition (i.e., “recovery period”). This research suggested that this elevated intake of fat might be considered a ‘reward’ post competition for these athletes [2,62]. Greater education on match day nutrition strategies are required to optimise energy and carbohydrate intake prior to competition. Additionally, in many sports athletes need to compete multiple times a week therefore it is integral that recovery tactics aim to restore muscle glycogen within 24–48 h post-competition [57]. In these instances, it is essential to consume a carbohydrate and protein-rich meal shortly after the game, followed by another carbohydrate-rich meal two hours later to accelerate glycogen resynthesis [11].

### 4.3. Limitations

Studies in this systematic review included small numbers of participants and may not be generalisable to team sport disciplines more broadly. In addition, the heterogeneity of the included studies led to an inability to compare results across all studies and as a result a meta-analysis of data was not possible. Underreporting is an important consideration when assessing dietary intake; however, suboptimal intakes should not be attributed solely to underreporting and dietary assessment should encompass a range of influencing factors (i.e., body composition, appetite, nutrition knowledge etc.) [3]. Many studies explored the existence of intentional and unintentional underreporting, thus the findings of these analyses should be interpreted with caution.

### 4.4. Conclusions

This systematic review found that despite the publication of high-quality research studies, expert consensus statements and recognition of the consequences of inadequate intakes, team sport athletes’ total energy and carbohydrate intakes did not meet sports nutrition recommendations (i.e., IOC, ISSN, ASCM and sports specific research) for energy and carbohydrate. In contrast, many athletes met or exceeded recommendations for protein and/or fat. Further research into the development of sport-specific recommendations for energy and macronutrients in particular carbohydrate would be beneficial to further optimise distribution throughout a training week. Furthermore, nutrition in team sport environments requires a knowledge base of the physiological demands of training and competition, and therefore sports dietitians should work collaboratively with sports science teams when tailoring nutrition advice to meet energy and macronutrient needs. Future research is required to explore the factors that influence athletes’ dietary intakes.

## Figures and Tables

**Table 1:** Participants, Intervention, Comparison, Outcome and Study (PICOS) criteria for inclusion and exclusion of studies.

Parameter	Description
Population	Professional and semi-professional team sport athletes
Intervention OR exposure	Baseline dietary intake
Comparison	Dietary intake in comparison to sports nutrition guidelines and recommendations.
Outcomes	Meeting/not meeting sports nutrition guidelines and recommendations
Study design	RCT (where baseline dietary intake data available), cross-sectional, longitudinal, thesis (unpublished and published)

Abbreviations: RCT Randomised Control Trial.

**Table 2:** Table of keywords.

Concept	Keywords
Sport OR team sport	“sport*” OR “team sport*” OR “football” OR “soccer” OR “football” OR “netball” OR “AFL” OR “Aussie rules” OR “rugby” OR “basketball” OR “grid iron” OR “American football” OR “hockey”
Dietary intake OR food intake	“nutrient requirement*” OR “dietary intake” OR “daily food intake” OR “food intake”

**Table 3:** Eligibility criteria.

Inclusion	Exclusion
1. Studies that include only team sport athletes.	1. Studies that include only individual athletes or sports.
2. Studies published since 2012.	2. Studies published up to 2011.
3. Studies that include only professional or semi-professional team sport athletes.	3. Studies that include only non-professional or amateur team sport athletes.
4. Studies that include only adult team sport athletes (i.e., >18 years of age).	4. Studies that include adolescent and child team sports athletes (i.e., <18 years of age).
5. English language studies.	5. Non-English language studies.
6. Studies that include quantitative measures of dietary intake that can be converted into units of intake per day for each nutrient.	6. Studies that include nutrition habits, attitudes, educational strategies, knowledge, where dietary intakes cannot be compared.
7. Studies that include the dietary assessment of total energy carbohydrate, protein fat, micronutrient intake (i.e., iron (mg/day), calcium (mg/day), folate etc.).	7. Studies where only supplement or antioxidant intake is assessed.
8. Studies that assess dietary intake using a validated method of assessment (i.e., 7 day food diary, 7 day weighed food diary, food records, 3 day food diaries, FFQ, diet histories etc.) and therefore estimates absolute dietary intake.	8. Studies that assess dietary intake, however methods used provide dietary assessments represented in food groups, percentage of total energy etc.
9. Only human studies that include; RCT (where baseline dietary intake data available), cross-sectional, longitudinal, thesis.	9. No animal studies, RCT (where only post intervention dietary intakes available), conference posters, reviews, abstracts and web-based articles.
10. Published and unpublished research (i.e., thesis).	

Abbreviations: FFQ Food Frequency Questionnaire, RCT Randomised Control Trial.

**Table 4:** Reported energy, macronutrient and micronutrients intakes of male and female professional and semi-professional team sport athletes.

References	Participant Characteristics							Dietary Intake Data									
Author, Year	Sport	Country	Level	*N* (Gender)	Total Mass (kg)	Lean Mass (kg)	BF%	Energy Intake Reported (Estimated MJ Were Available)	CHO (g)	CHO (g·kg^−1^·day^−1^)	PRO (g)	PRO (g·kg^−1^·day^−1^)	Fat (g)	Fat (% TE)	Calcium (mg·day^−1^)	Iron (mg·day^−1^)	Quality Rating
Anderson et al. [23], (2017)	Football (soccer)	England	P	*n* = 6 (male)	80.5 ± 8.7	11.9 ± 1.2	65.0 ± 6.7	3186 ± 585 kcal·day^−1^ (13.3)	330 ± 98	4.2 ± 1.4	205 ± 30						+
Andrews and Itsiopoulus [25] (2016)	Football (soccer)	Australia	P, SP	*n* = 73 (male)	P 79.6 ± 7.7 SP 75.6 ± 7.6			P 11,525 ± 1987 kJ·day^−1^ SP 10,831 ± 3842 kJ·day^−1^ (P 11.5 ± 2.0, SP 10.8 ± 3.8)	P 302.4 ± 72.3 SP 298.7 ± 148.5	P 3.5 ± 0.8 SP 3.9 ± 1.8	P 152.3 ± 27.7 SP 149.1 ± 46.8	P 1.9 ± 0.3 SP 2.0 ± 0.6	P 95.9 ±31.7 SP 85.8 ±37.8	P 30.4 ± 7.3 SP 29.5 ± 7.4			∅
Bettonviel et al. [24] (2016)	Football (soccer)	Netherlands	P	*n* = 14 (male)	77.0 ± 8.6			2988 ± 583 kcal·day^−1^ (12.5)	365 ± 76	4.7 ± 0.7	145 ± 24	1.9 ± 0.3	97 ± 26	29.0 ± 3.6			∅
Bilsborough et al. [26] (2016)	Australian Football	Australia	P	*n* = 45 (male)	86.8 ± 7.9	[7.9 ± 1.8–10.5 ± 2.7]	[71.2 ± 6.8–76.8 ± 6.4]	[11.1–13.9]	[321 ± 142–379 ± 66]	4.1 ± 1.6	[118 ± 67–215 ± 60]	1.9 ± 0.9	[61 ± 29–110 ± 40]	[19.0 ± 5.0–33.1 ±6.7]			∅
Bradley et al. [27] (2015)	Rugby Union	Europe (non-specified)	P	*n* = 14 (male)	F 110 ± 6.2 B 93.6 ± 5.9			F 16.6 ± 1.25 MJ·day^−1^ B 14.2 ± 1.20 MJ·day^−1^		F 3.5 ± 0.8 B 3.4 ± 0.7		F 2.7 ± 0.5 B 2.7 ± 0.3		F 32 B 33	1733 ± 694	24 ± 9	∅
Bradley et al. [10] (2015)	Rugby Union	Europe (non-specified)	P	*n* = 20 (male)	F 109.3 ± 6.9 B 91.7 ± 6.6	F 13 ± 3 B 9.3 ± 2	F 94.9 ± 4.5, B 83.1 ± 5.4	F 14.8 ± 1.9 MJ·day^−1^ B 13.3 ± 1.9 MJ·day^−1^		F 3.3 ± 0.7 B 4.14 ± 0.4		F 2.52 ± 0.3 B 2.59 ± 0.6					∅
Conejos et al [28] (2011)	Football (soccer)	Spain	P	*n* = 22 (male)				Fwd. 12.7 ± 2.9 MJ·day^−1^ M 14.0 ± 6.2 MJ·day^−1^ D 14.8 ± 2.6 MJ·day^−1^ G 12.2 ± 4.6 MJ·day^−1^	Fwd. 342.5± 92.9 M 382.1 ± 187.2 D 419.1 ± 98.3 G 320.3 ± 11.9		Fwd. 138.7 ± 27.5 M 144.8 ± 56.9 D 144.5 ± 19.9, G 142.8 ± 100.1		Fwd. 120.3 ± 59.6 M 131.6 ± 62.6 D 124.5 ± 36.1 G 109.8 ± 45.3	Fwd. 34.7 M 35.6 D 32.2 G 34.9	Fwd. 1361.5 ± 549.0 M 1592.3 ± 966.1 D 1208.4 ± 457.1 G 1499.7 ± 1035.9	Fwd. 13.4 ± 3.7 M 18.9 ± 9.7 D 22.6 ± 6.8 G 15.5 ± 10.2	∅
Devlin et al. [43] (2017)	Australian Football (AF) Football (Soccer, F)	Australia	P, SP	*n* = 66 AF = 48, F = 18 (male)	AF (P) 87.8 ± 9.2 AF (SP) 82.9 ± 9.0 F (P) 75.6 ± 5.6	AF (P) 15.1 ± 2.4, AF (SP) 16.7 ± 2.7 F (P) 12.8 ± 1.9	AF (P) 65.4 ± 7.9 AF (SP) 61.2 ± 3.9 F (P) 56.8 ± 5.2	AF (P) 17.3 ± 4.2 MJ·day^−1^ AF (SP) 13.2 ± 2.5 MJ·day^−1^ F (P) 9.4 ± 2.3 MJ·day^−1^	AF (P) 406 ± 132 AF (SP) 368 ± 93 F (P) 220 ± 76	AF (P) 4.6 ± 1.5 AF (SP) 4.5 ± 1.2 F (P) 2.9 ± 1.1	AF (P) 295 ± 97 AF (SP) 171 ± 52 F (P) 140 ± 35	AF (P) 3.4 ± 1.1 AF (SP) 2.1 ± 0.7 F (P) 1.9 ± 0.5		AF (P) 29 ± 6 AF (SP) 28 ± 8 F(P) 33 ± 9			∅
Grams et al. [29] (2016)	Wheelchair basketball	Spain	P	*n* = 17 (male)	75.5 ± 13.5			2673 ± 485 kcal·day^−1^ (11.2)		3.9 [Range: 1.8–8.1]		1.7 ± 0.6		33.7 ± 5.5			+
Gravina et al. [30] (2012)	Football (soccer)	Spain	P, SP	*n* = 28 (female)	61 ± 8.4	16.7 ± 3.2		2271 ± 578 MJ·day^−1^ (9.5)						33 ± 7			+
Hidalgo et al. [31] (2015)	Football (soccer)	Mexico	P	*n* = 6 (male)	68.3 ± 2.0		32.5 ± 1.0	3042 ± 56 kcal·day^−1^ (12.7)	364 ± 17.5	5.4 ± 0.3	145 ± 14	2.2 ± 0.2	113 ± 6.3	33 ± 0.0			∅
Jenner et al. [3] (2018)	Australian Football	Australia	P	*n* = 46 (male)	86.3 ± 9.4	10.8 ± 2.3	73.9 ± 9.1	9.1 ± 1.8 MJ·day^−1^	201 ± 56	2.4 ± 0.8	150 ± 30	1.8 ± 0.4	78.9 ± 20.0	32 ± 4.5	952 ± 287		∅
Kirwan et al. [32] (2012)	American Football	America	SP	*n* = 15 (male)	93.8 ± 15.3		70.5 ± 7.7	3518 ± 849 kcal·day^−1^ (14.7)	353 ± 118		169 ± 52		160 ± 45				∅
MacKenzie et al. [33] (2015)	Rugby Union	Australia	P	*n* = 25 (male)	100.2 ± 13.3		77.0 ± 7.0	13605 ± 3639 kJ·day^−1^ (13.6)	352 ± 115	3.6 ± 1.3	211 ± 62	2.2 ± 0.7	101 ± 34	28 ± 5.0			∅
Molina-lopez et al. [36] (2013)	Handball	Spain	P	*n* = 14 (male)	86.7 ± 5.4	11.6 ± 2.5		2974.5 ± 211.1 kcal·day^−1^ (12.4)	360.9 ± 27.6	4.2 ±0.4	133.4 ±14.3	1.5 ± 0.2	118.6 ± 22.5		1251.4 ± 338.2	24.2 ± 8.5	∅
Ono et al. [34] (2011)	Football (soccer)	England	P	*n* = 24 (male)				[2648-4606 kcal·day^−1^] (11.1–19.3)	505.2 ± 120		141.7 ± 22.8						∅
Potgieter, et al. [35] (2014)	Rugby Union	South Africa	SP	*n* = 11 (male)	95.5 ± 13.6	18.2 ± 5.7	79.7 ± 9.0	45.4 ± 9.0 kcal·kg^−1^·day^−1^		4.3 ± 0.4		2.4 ± 0.7		33.8 ± 4.3	1250 ± 403		∅
Raizel et al. [37] (2017)	Football (soccer)	Brazil	P	*n* = 19 (male)	71.8 ± 8.2	4.9 ± 1.5		40.74 ± 12.8 kcal·kg^−1^·day^−1^		5.4 ± 1.9	136.4 ± 57.4	1.9 ± 0.8	91.2 ± 35.9				∅
Tooley et al. [2] (2015)	Rugby League	UK	P	*n* = 10 (male)	97.3 ± 3.1			3731 ± 164 kcal·day^−1^ (15.6)	467 ± 24	4.9 ± 0.3	209 ± 10	2.2 ± 0.2	124 ± 10	30 ± 1.0			∅
Valliant et al. [38] (2012)	Volleyball	America	SP	*n* = 11 (female)	75.4 ± 13.4	24.5 ± 5.9		1756 ± 557.5 kcal·day^−1^ (7.3)	224.3 ± 64.4	3.08 ± 1.1	69.3 ± 26.8	0.9 ± 0.3	67.4 ± 27.8	33.7 ± 6.4			∅
Vermeulen et al. [39] (2017)	Ice-hockey	Canada	SP	*n* = 25 (female)	67.0 ± 1.7			2354 ± 74 kcal·day^−1^ (9.8)	305 ± 15	4.6 ± 0.2	91 ± 4	1.4 ± 0.1	82 ± 4	32 ± 1.0	1041 ± 64	19 ± 3	∅

Abbreviations: AF Australian Football, F Football (soccer), BF% Body fat percentage, CHO Carbohydrate, D Defenders, Fwd Forwards, GK Goal-keepers, kcal·day^−1^ Calories per day, Kcal·kg^−1^·day^−1^ Calories per kg per day, kJ·day^−1^ Kilojoules per day, MJ·day^−1^ Mega joules per day, M Midfielders *PRO* Protein, *P* Professional, *SP* Semi-professional. Quality rating criteria: Positive (+), Neutral (∅) and Negative (−).
